# A comprehensive analysis of cotton *VQ* gene superfamily reveals their potential and extensive roles in regulating cotton abiotic stress

**DOI:** 10.1186/s12864-020-07171-z

**Published:** 2020-11-16

**Authors:** Pengyun Chen, Fei wei, Shuaishuai Cheng, Liang Ma, Hantao Wang, Meng Zhang, Guangzhi Mao, Jianhua Lu, Pengbo Hao, Adeel Ahmad, Lijiao Gu, Qiang Ma, Aimin Wu, Hengling Wei, Shuxun Yu

**Affiliations:** 1 State Key Laboratory of Cotton Biology, Institute of Cotton Research of Chinese Academy of Agricultural Sciences, Anyang, 455000 Henan China; 2 School of Life Science, Zhengzhou University, Zhengzhou, 450000 China; 3 College of Agronomy, Northwest A&F University, Yangling, 712100 China

**Keywords:** *Gossypium*, Valine glutamine (VQ), Phylogenetic, Expression analysis

## Abstract

**Background:**

Valine-glutamine (*VQ*) motif-containing proteins play important roles in plant growth, development and abiotic stress response. For many plant species, the *VQ* genes have been identified and their functions have been described. However, little is known about the origin, evolution, and functions (and underlying mechanisms) of the *VQ* family genes in cotton.

**Results:**

In this study, we comprehensively analyzed the characteristics of 268 *VQ* genes from four *Gossypium* genomes and found that the *VQ* proteins evolved into 10 clades, and each clade had a similar structural and conservative motif. The expansion of the *VQ* gene was mainly through segmental duplication, followed by dispersal. Expression analysis revealed that many *GhVQs* might play important roles in response to salt and drought stress, and *GhVQ18* and *GhVQ84* were highly expressed under PEG and salt stress. Further analysis showed that *GhVQs* were co-expressed with *GhWRKY* transcription factors (TFs), and microRNAs (miRNAs) could hybridize to their cis-regulatory elements.

**Conclusions:**

The results in this study broaden our understanding of the *VQ* gene family in plants, and the analysis of the structure, conserved elements, and expression patterns of the *VQs* provide a solid foundation for exploring their specific functions in cotton responding to abiotic stresses. Our study provides significant insight into the potential functions of *VQ* genes in cotton.

**Supplementary Information:**

**Supplementary information** accompanies this paper at 10.1186/s12864-020-07171-z.

## Background

The *VQ* genes form a large gene family with important roles in growth, development and abiotic stress tolerance in plants [1–3]. The VQ proteins have a conserved VQ motif [F**hVQ*hTG (F, phenylalanine; *, any amino acid; h, hydrophobic residue; V, valine; Q, glutamine; T, tryptophan; G, glycine)] [4, 5] and interact with WRKY TFs via the conserved residues V and Q. In *Arabidopsis thanliana*, many *VQ* genes have been reported to function in development and responses to abiotic stress. For example, *AtVQ23* (sigma factor-binding protein 1, *SIB1*), and *AtVQ16* (*SIB2*) were found to interact with *AtWRKY33* to increase the resistance of *Arabidopsis* plants to *Botrytis cinerea* [6]. In another study, *AtVQ16* and *AtVQ23* have also been proven could interact with *AtWRKY57*, and *AtVQ16* and *AtVQ23* can enhance the competitions on *AtWRKY57* to *AtWRKKY33* in regulating *JASMONATE ZIM-DOMAIN1* (*JAZ1*) and *JAZ5* [7]. Moreover, *JASMONATE-ASSOCIATED VQ MOTIF GENE1* (*JAV1/AtVQ22*) has been addressed to be as a key negative regulator of the jasmonate signalling [8]. For instance, *AtVQ09* acts as a repressor of *AtWRKY08* factor to modulate salt tolerance in *Arabidopsis* [9]. Recently, *MdVQ10* and *MdVQ15* were also described to interact with *MdWRKY52* to regulate pathogen defense and development in apple (*Malus domestica Borkh*) [10]. In addition, *OsVQ7* interacts with *OsWRKY24* and play roles in NO signaling contributing to the tolerance of various stresses and development in rice (*Oryza sativa*) [11].

*VQs* have also been reported to perform other functions. It has been demonstrated that *AtVQ14* (*HAIKU*, *IKU1*) interacts with *MINISEED3* (*MINI3*, *AtWRKY10*) to reduce the expression of *IKU2*, affecting the seed size [1], and *AtVQ20* regulates the male gametogenesis in *Arabidopsis* [12]. In addition, some *VQs* have been shown to interact with ETHYLENE RESPONSE FACTORs (*ERF*), mitogen-activated protein kinases (*MAPKs*), and miRNAs in response to environmental stresses in plants [13, 14]. Otherwise, some abiotic stress-related genes have been isolated from *Arabidopsis* and other plants [15–19], including *VQ* TFs, *WRKY* TFs, and other TFs.

Cotton is an important, widely cultivated fiber and oil crop that is essential for the textile industry and provides nutrient-rich edible oil [20]. Various biotic and abiotic stresses, including pathogen infection, drought and salinity stresses, consistently and severely affect the formation of cotton production [21–23]. Therefore, it is very important to develop new cultivars with high resistance to biotic and abiotic stresses. Recently, *VQ* family genes have been identified at genome-wide levels in several plants, including *Arabidopsis* (34 *VQs*) [24], soybean (*Glycine max*) (74 *VQs*) [25], rice (39 *VQs*) [26], maize (*Zea mays*) (61 *VQs*) [27], Chinese cabbage (*Brassica rapa spp. pekinensis*) (57 *VQs*) [28], apple (49 *VQs*) [10], tea (*Camellia sinensis*) (25 *VQs*) [29], tomato (*Solanum lycopersicum*) (26 *VQs*) [30], sunflower (*Helianthus annuus*) (20 *VQs*) [31], tobacco (*Nicotiana tabacum*) (59 *VQs*) [19], *Cicer arietinum* (19 *VQs*) [32] and *Medicago truncatula* (32 *VQs*) [32]. However, the genomic information and genetic evolution relationships of *VQs* are not clear in *Gossypium* spp*.*, and the expression patterns of these genes in different tissues and in responses to abiotic stresses remain unknown. The released for cultivated *Gossypium* spp. (*G. hirsutum* Linn., *G. barbadense* L., *G. raimondii* Ulbr and *G. arboretum* L.) genome sequences and their annotation have brought much convenience for thoroughly investigation of their genomics and genetic information [33–35]. In this study, using the annotations of four *Gossypium* genomes, we identified the *VQs* of these *Gossypium* species, performed the analyses of phylogeny, conserved structural motifs, whole-genome duplication (WGD) and functional interaction networks of the *VQs*, and predicted the microRNA target profiles of the *VQs*. The comprehensive analysis of the *VQ* gene family in *Gossypium* will contribute to identify new key candidate genes for diverse stress resistance in cotton breeding.

## Results

### Identification and comparative analysis of *VQs* in plants

To identify VQ family in *G. hirsutum*, *G. barbadense*, *G. raimondii* and *G. arboretum*, the AtVQ proteins were used as query sequences to search against the protein databases of the four *Gossypium* species, and the VQ-domain Pfam (PF05678) was also applied. In total, 89 *GhVQs*, 89 *GbVQs*, 45 *GrVQs*, and 45 *GaVQs* were identified and named in *G. hirsutum*, *G. barbadense*, *G. raimondii* and *G. arboretum*, respectively (Additional File 1, Supplemental Table S1). In addition, the physiological and biochemical properties of 268 *VQs* in *Gossypium* species were determined, including CDS length, GC count, isoelectric point (pI) and molecular weight (MW) (Additional File 1, Supplemental Table S1). The CDS lengths of these *Gossypium VQs* ranged from 279 bp (*GhVQ89* and *GbVQ89*) to 1443 bp (*GbVQ15*), the average GC content of the transcripts was 46.01, their exon numbers varied from 1 to 9, and only a small percentage of *VQs* contained introns (3.37% *GhVQs*, 3.37% *GbVQs*, 6.67% *GaVQs*, and 31.11% *GrVQs*). The pI values varied from 4.159 (*GbVQ33* and *GbVQ78*) to 11.496 (*GhVQ07*) and the MW values ranged from 10.346 kDa (*GbVQ89*) to 52.058 kDa (*GbVQ15*) (Additional File 2, Supplemental Fig. S1).

**Fig. 1:**
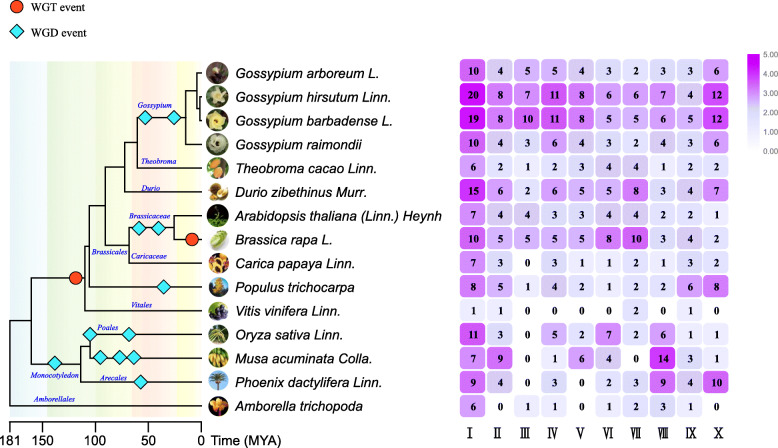
The *VQ* gene family evolutionary relationship and the number details of the 15 plant species. The left of this figure shows the evolutionary relationships of the species; the right of this figure shows the number details of the *VQ* family of each group

To perform comparative genomic analyses, we searched another 11 plant species for VQ proteins. The evolutionary relationships of the species and the number of their *VQ* genes are shown in Fig. 1. The data revealed that the numbers of *VQs* in *A. trichopoda*, *P. dactylifera*, *V. vinifera*, *P. trichocarpa*, and *T. cacao* were less than those in the four *Gossypium* species (Additional File 3, Supplemental Table S2). The comparative structure analysis of *VQs* showed that almost all the *VQs* had a few introns and encoded relatively small proteins, and only 3 *GhVQs*, 3 *GbVQs*, 3 *GaVQs* and 14 *GrVQs* had more than one intron. We speculated that the WGD events that occurred during the evolution of angiosperms increased the numbers of the *Gossypium VQs*, and these events helped the *VQs* to gain new functions through neofunctionalization. However, the evolutionary forces that shaped the current intron/exon gene structures remain unknown.

### Phylogenetic analysis of VQs

**Fig. 2:**
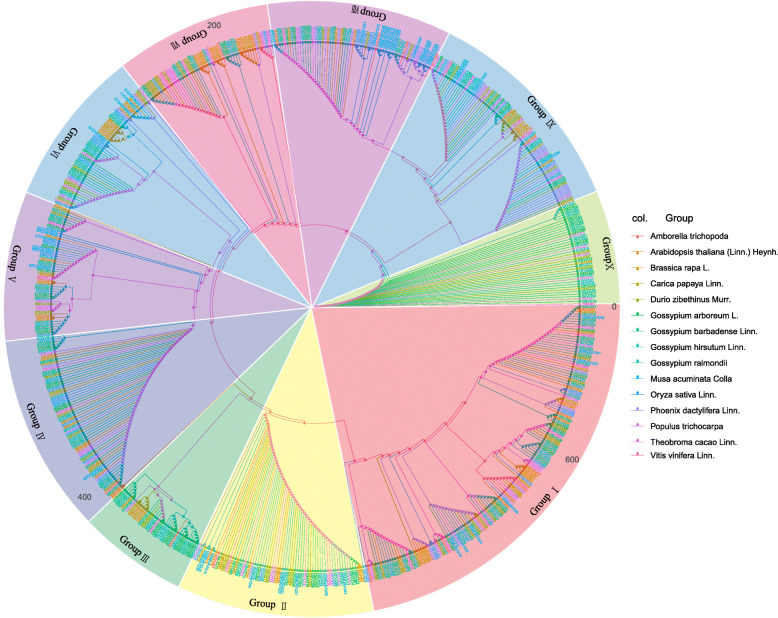
The *VQ* gene family phylogenetic relationship of the 15 plant species. The Phylogenetic tree includes 268 *VQs* from *Gossypium* and 388 *VQs* from other 11 plants. Maximum likelihood (ML) bootstrap values are shown in the major nodes

To explore the relationships among VQs in *Gossypium*, we conducted a phylogenetic analysis of the VQs from the 15 plant species (Fig. 2), and a phylogenetic tree between *Gossypium* spp. and *Arabidopsis* was also constructed (Additional File 4, Supplemental Fig. S2). The tree contained 656 VQs. These proteins were divided into 10 clades based on the nomenclature of the *Arabidopsis* VQs. The largest group (Group I) contained 20 GhVQs, 20 GbVQs, 10 GaVQs, and 10 GrVQs. Group IX was the smallest group, including 4 *GhVQs*, 5 *GbVQs*, 3 *GaVQs* and 3 *GrVQs*. Previous research has verified that VQ proteins contain a conserved motif composed of F**hVQ*hTG [4, 5]. In our study, among the 656 VQs, 212 proteins (in Group I and Group II) had the amino acid “M” next to “VQ” (simple M-VQ model); 159 proteins (in Group III, Group IV, and Group VII) had the amino acid “V” next to “VQ” (simple V–VQ model); and 285 proteins (in Group V, Group VI, Group VII, Group IX and Group X) had the amino acid “L” next to “VQ” (simple L-VQ model) (Additional File 5, Supplemental Fig. S3). The VQs with rarer amino acids of the *Gossypium* species were also scattered in Group I to Group X, and the clusters of VQs were similar to those in angiosperms [3].

### Cis-regulation elements and structural composition of the *VQs*

**Fig. 3:**
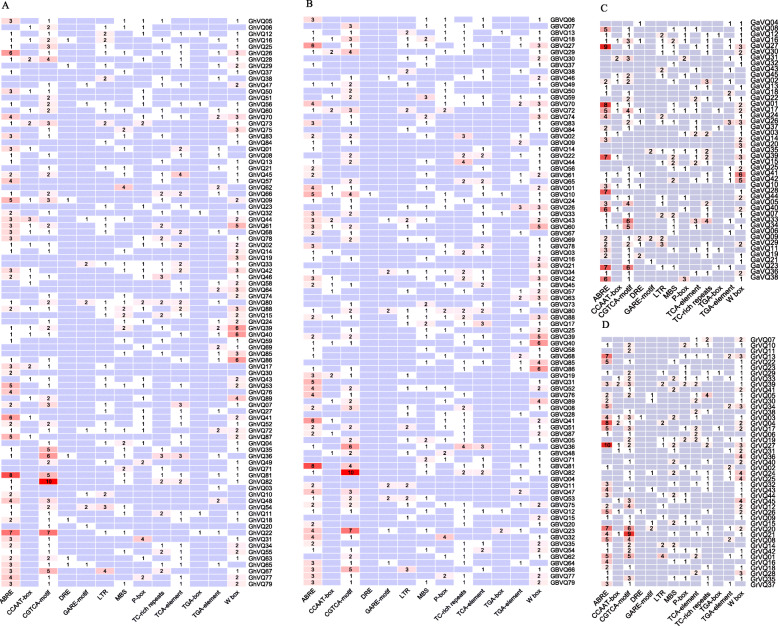
Potential cis-elements in promoters of *VQs* in *Gossypium*. **a**, **b**, **c**, **d** are the identified cis-elements of *GhVQs*, *GbVQs, GaVQs and GrVQs*, respectively

The cis-regulation elements in the promoters (from 2000 bp to − 1 bp) of the four *Gossypium VQs* were analyzed using the PlantCARE tool. 715, 701, 386 and 399 cis-regulation elements from the *GhVQs*, *GbVQs*, *GaVQs* and *GrVQs* were identified, respectively. Among these, seven kinds of hormone-responsive cis-regulation elements, ABRE (ABA-responsive element), P-box, TGA-box, TGA-element, TCA-element, CGTCA-motif, and GARE-motif were associated with ABA, ethylene, salicylic acid (SA), methyl jasmonate (MeJA), auxin (IAA), and gibberellin (GA), respectively; and six regulatory elements, MBS, TC-rich repeats, LTR, DRE-motif, W-box, and CCAAT-box were related to drought, cold stress, and pathogen defense (Fig. 3). Moreover, the promoters of 66 *GhVQs*, 69 *GbVQs*, 36 *GrVQs* and 34 *GaVQs* possessed WRKY-binding sites (W-box) (Fig. 3). The diversity of the cis-regulation elements in the promoters of *VQs* indicated that *VQs* might participate in regulating the *Gossypium* response to endogenous hormones and diverse environmental stimuli.

**Fig. 4:**
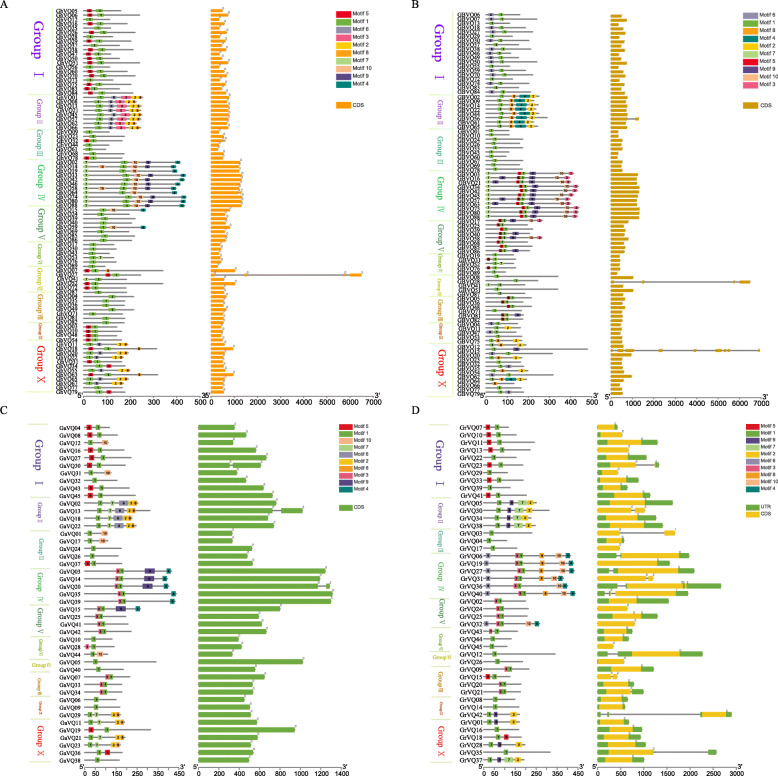
The converted motif and gene structure of *VQs* in *Gossypium*. **a**, **b**, **c**, **d** are the converted motif and gene structure of *GhVQs*, *GbVQs*, *GaVQs* and *GrVQs*, respectively

Motif compositions and exon-intron structures of the *VQs* were shown in Fig. 4. Combining the phylogenetic data groups of the four *Gossypium VQs*, we found that there were more motif types in Group IV, including Motif 1, Motif 4, Motif 7, Motif 9, and Motif 10; and followed by Group II, containing Motif 1, Motif 2, Motif 3, Motif 6, and Motif 8. Not surprisingly, Motif 1 existed in almost all of the *VQs*, suggesting that it was the most conservative motif. The differences in motif composition among the four *Gossypium VQs* suggested that they might perform different functions in diverse *Gossypium* species. Most of the *VQs* had no intron, including 96.63% (86/89) of *GhVQs*, 96.63% (86/89) of *GbVQs*, 68.89% (31/45) of *GrVQs*, and 93.33% (42/45) of *GaVQs*. The remaining *VQs*, which were widely distributed in Group VII and Group X, contained one to eight introns. In general, *VQs* in the same clades would share similar motif elements and structural compositions, indicating that the *VQ* members in the same subgroup could have similar functions.

### Chromosomal distribution, synteny and duplication analysis

**Fig. 5:**
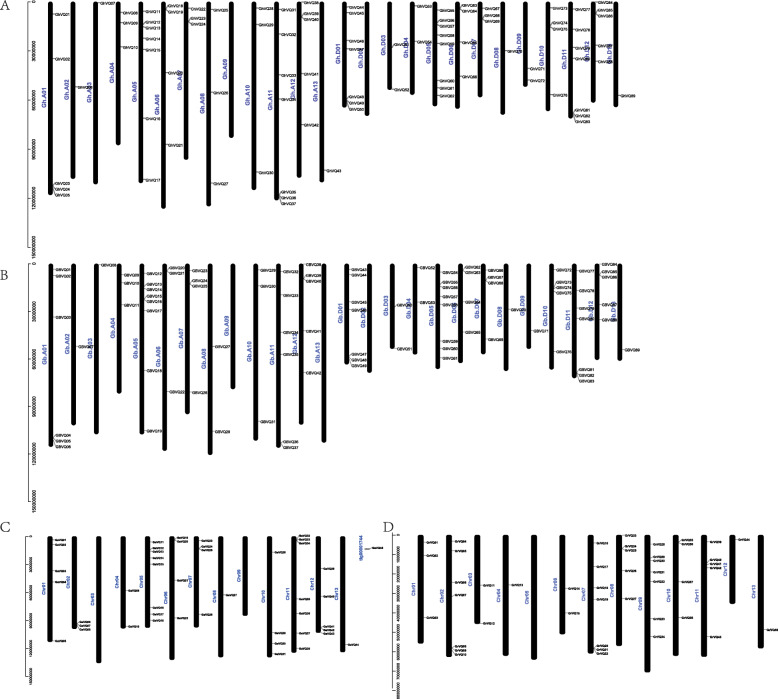
Distribution of the *VQs* on chromosomes. **a** The *89 GhVQs* distributed in *G. hirsutum*. **b** The 89 *GbVQs* distributed in *G. barbadense*. **c** The 45 *GaVQs* distribution in *G. arboreum*. **d** The 45 *GrVQs* distribution in G. *raimondii*

In this study, *VQs* were detected located in most chromosomes with a few exceptions. For example, Gh_A09 and Gh_D02 were in *G. hirsutum*, Gb_A09, Gb_A13 and Gb_D02 were in *G. barbadense*, Ga_Chr03 and Ga_Chr09 in *G. arboretum*, and Gr_Chr05 were *G. raimondii* (Fig. 5). For the two allotetraploid species of *Gossypium*, Gh_D05 (eight genes/~ 9%), Gb_A05 (eight genes / ~ 9%), and Gb_D05 (eight genes / ~ 9%) contained more *VQ* genes than other chromosomes, while Gh_A02, Gh_A03, Gh_A13, Gh_D08, Gh_D13, Gb_A02, Gb_A03, Gb_D08, Gb_D09, and Gb_D13 only contained one gene. For the two diploid species, Ga_Chr05 (eight genes / ~ 17.8%), Gr_Chr02 (seven genes / ~ 17.8%), Gr_Chr07 (seven genes / ~ 17.8%) and Gr_Chr09 (seven genes / ~ 17.8%) contained more *VQs*, and Ga_Chr08, Ga_Chr13, Gr_Chr04, Gr_Chr12, and Gr_Chr13 only contained one gene. Most *VQs* in the four *Gossypium* species were distributed at both ends of the chromosomes, which corresponded to the position of the telomere.

**Fig. 6:**
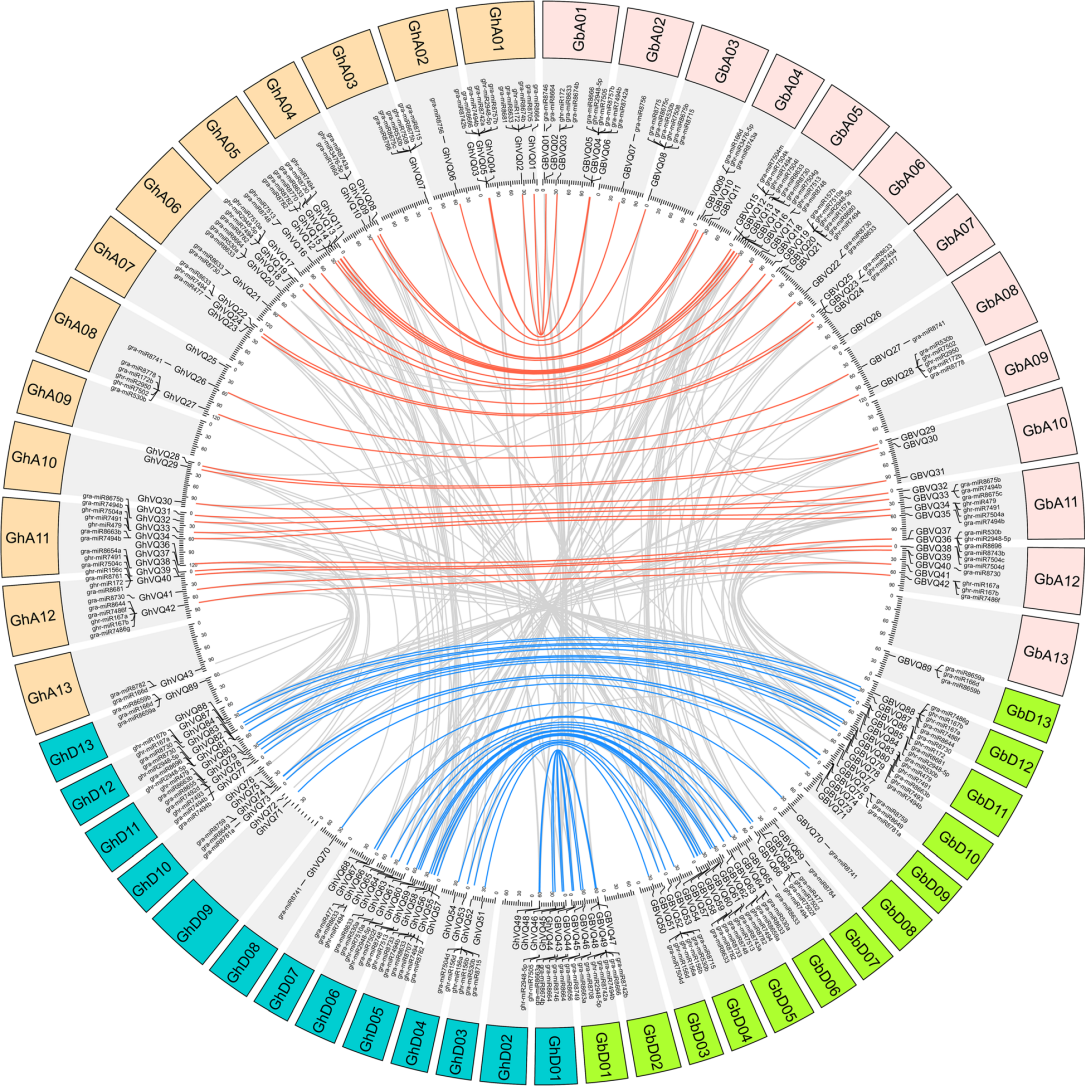
Ortholog and paralog pairs of *VQs* in *G. hirsutum* and *G. barbadense*. The lines regarding orthologous gene pairs are colored by blue and orange, and the paralogous gene pairs are colored by grey lines. The central links are predicted miRNA target genes

The orthologous *VQs* were first identified between *G. hirsutum* and *G. arboreum* with *G. raimondii*. A total of 83 *GhVQs* were orthologous genes in the two-diploid *Gossypium*, of which 40 gene pairs showed A genome origin, while 43 gene pairs showed D genome origin (Additional Files 6 and 7, Supplemental Fig. S4 and S7, Table S3). Subsequently, orthologous gene identification was also conducted between *G. barbadense* and *G. arboreum* with *G. raimondii*, and there were 84 orthologous *GbVQs*. Of which, 40 gene pairs showed A genome origin, while 44 gene pairs showed D genome origin (Additional Files 7 and 8, Supplemental Fig. S5 and S3). Orthologous genes between *G. hirsutum* and *G. barbadense* were also identified. It was found that 39 gene pairs in Gh_At and Gb_At subgenomes and 42 gene pairs in Gh_Dt and Gb_Dt subgenomes (Fig. 6 and Additional File 7, Supplemental Table S3). In addition, *GhVQ27*, *GhVQ61*, *GhVQ68*, *GbVQ28*, *GbVQ60* and *GbVQ67* had no orthologous genes in the diploid *Gossypium* species.

As previously described, duplication contributed to the expansion of genes in the polyploid events in plants [36]. The tetraploid species *Gossypium* of have undergone a genome duplication since their diverge from the two diploid species of *Gossypium*. In our study, we have identified the *VQ* duplication event, and the WGD/segmental event likely contributed to the expression regulation of *VQs* in *Gossypium*. The percentages of *VQs* derived from WGD were 60.47% in the At-subgenome of *G. hirsutum*, 63.04% in the Dt-subgenome of *G. hirsutum*, 69.04% in the At-subgenome of *G. barbadense*, 59.57% in the Dt-subgenome of *G. barbadense*, 62.22% in *G. raimondii*, and 57.78% in *G. arboretum* (Additional File 9, Supplemental Table S4). Gene duplication events after the divergence of *Gossypium* species resulted in a high number of paralogous genes in both allotetraploid *Gossypium* species.

### Prediction of miRNA target sites

miRNA had been predicted to target the *VQ* genes in *Arabidopsis* [37, 38] and tea [39]. To determine the miRNA-mediated post-transcriptional regulation of *VQs* in two allotetraploid species of *Gossypium*, we predicted the target sites of miRNAs in the coding (CDS) regions of the *GhVQs* and *GbVQs*. In *G. hirsutum*, 46 sites of 34 *GhVQs* were detected that could be targeted by 22 miRNAs, while 46 sites of 32 *GbVQs* could be targeted by 21 miRNAs (Fig. 6 and Additional File 10, Supplemental Table S5). Of these, six *VQ* genes (*GhVQ02*, *GhVQ40*, *GhVQ86*, *GbVQ02*, *GbVQ40* and *GbVQ86*) were predicted to be targeted by Ghr-miR172 in the CDS regions; and six *VQ* genes (*GhVQ39*, *GhVQ52*, *GhVQ85*, *GbVQ39*, *GbVQ51* and *GbVQ85*) were targeted by Ghr-miR156 (Ghr-miR156a, Ghr-miR156b, Ghr-miR156c and Ghr-miR156d) at 10 prediction sites. Ghr-miR172 and Ghr-miR156 were reported to be involved in some biological processes, including the responses to developmental cues and abiotic stress in plants [40–42]. However, it requires further experiments to verify the regulation mechanism and functions of those predicted miRNAs and their targets in *Gossypium*.

### Expression pattern analysis and function verification

**Fig. 7:**
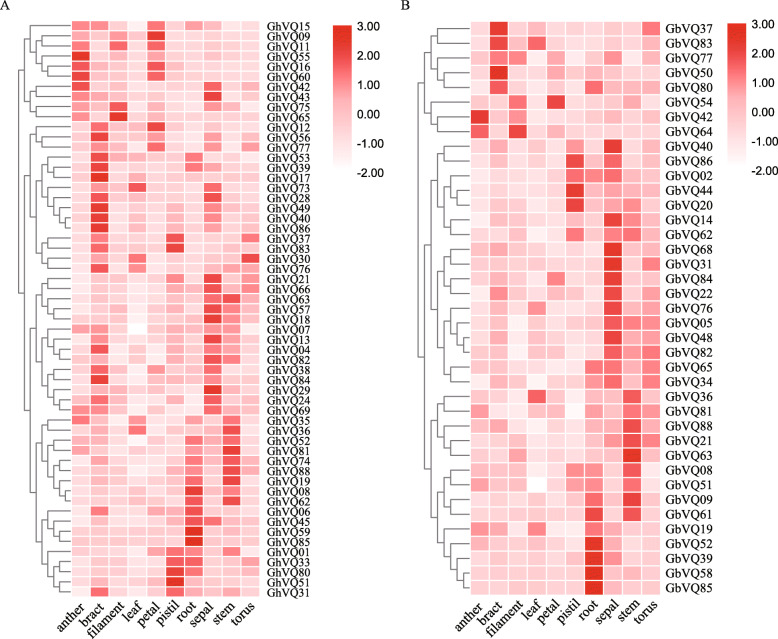
The expression of *Gossypium VQs* in different tissues. **a** The expression of the selected *VQs* in *G. hirsutum*; **b** The expression of the selected *VQs* in *G. barbadense*

Expression profiles of the *VQs* in the two allotetraploid kinds of *Gossypium* were analyzed with available transcriptome data (Additional Files 11 and 12, Supplemental Fig. S6 and S7 and Supplemental Table S6). In this study, the *GhVQs* and *GbVQs* with average FPKM values > 5 and being present in at least two samples were identified as potentially expressed transcripts. Fifty-seven *GhVQs* and 39 *GbVQs* were selected, and their expression profiles tested in 10 tissues, including the anther, bract, filament, leaf, petal, pistil, root, sepal, stem and torus (Fig. 7). For 57 *GhVQs*, 22 genes were highly expressed in roots and *GhVQ82* had the highest expression level, and 14 genes were highly expressed in leaves. There were only a few genes expressed in the anther, bract, filament, petal, pistil, sepal, stem and torus (Fig. 7a). For 39 *GbVQs*, 17 *GbVQs* were strongly highly expressed in roots, and 12 genes were strongly expressed in leaves (Fig. 7b). The different expression profiles of *VQ* genes suggest that they have different functions in distinct tissues and developmental stages.

**Fig. 8:**
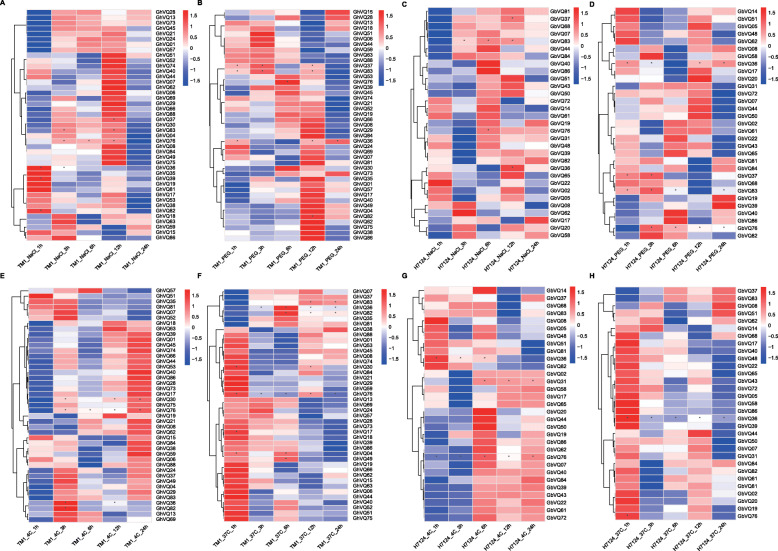
Stress-induced expression profiles of *GhVQs* and *GbVQs*. **T**he expression levels of 43 *GhVQs* and 31 *GbVQs* under salt (**a** and **c**), drought (**b** and **d**), cold (**e** and **g**) and heat (**f** and **h**) stresses. The colors varied from blue to red represent the scales of the relative expression levels

**Fig. 9:**
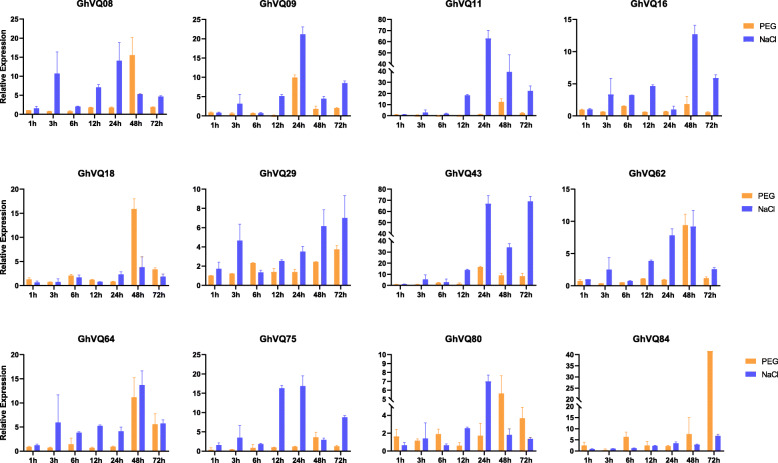
The qPCR relative transcriptional levels of *GhVQs* under different stresses. The time of posts of different treatments are shown on the x-axis, and the relative expression levels are shown on the y-axis

*VQs* were widely identified in the abiotic stress responses in angiosperms [3, 32]. In this study, the expression patterns of *VQs* in the allotetraploid *Gossypium* types under salt, drought, cold and heat stresses were analyzed using the published data. In total, 43 *GhVQs* and 37 *GbVQs* had different expression profiles under the four abiotic stress treatments (Fig. 8). Under salt stress, 29 *GhVQs* were significantly up-regulated at 12 h, and 21 *GbVQs* were up-regulated at 6 h (Fig. 8a and c). Upon PEG treatment, most of the *GhVQs* and *GbVQs* were highly expressed at 12 h (Fig. 8b and d). During cold stress, 33 *GhVQs* and 19 *GbVQs* were up-regulated at 24 h (Fig. 8e and g). Most of the *GhVQs* and *GbVQs* under the hot treatment were highly expressed at 1 h (Fig. 8f and h). To validate of the expression results of *GhVQs* in response to salt and drought stresses, we conducted qRT-PCR analyses of 12 *GhVQs* after treatments with PEG and salt treatment. In the presence of PEG, *GhVQ08*, *GhVQ18*, *GhVQ62*, *GhVQ64*, *GhVQ80*, and *GhVQ84* had high expression levels at 48 h, while these *GhVQs* except *GhVQ18* and *GhVQ84*, were highly expressed during 24–48 h under salt treatment (Fig. 9). The qRT-PCR results were slightly different from the RNA-seq data, but these findings suggest that some *GhVQs* were involved in plant response to drought and salt stresses.

### Co-expression and interaction networks of *GhVQs*

**Fig. 10:**
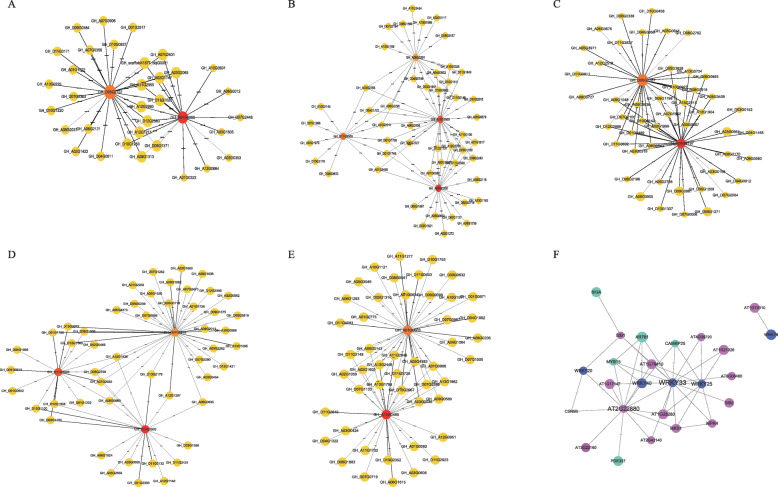
Co-expression and functional interaction networks of *GhVQs*. Orange circular ones are the *GhVQ* co-expression genes (**a**, **b, c**, **d** and **e**), while yellow ones are other co-expression genes. **f** The functional interacting network models of *GhVQs*. Homologous genes in *Gossypium* and *Arabidopsis* are shown in pink and blue, respectively

To understand the putative roles of *VQs* in plant adaptation to drought and salt stresses, we conducted a co-expression analysis. Ten *GhVQs* were found to co-express with another 227 functional genes (Fig. 10 and Additional File 13, Supplemental Table S7). Among these, six and seven *VQs* were identified in different modules of drought stress and salt stress, respectively, while *GhVQ37*, *GhVQ59* and *GhVQ83* were detected coexisting during the two stress treatments. Moreover, these 227 genes co-expressing with 10 *GhVQs*, contained multiple TFs, including domain AP2, bHLH, F-box, GRAS, p450, PLB03212, WD40 and WRKY (Fig. 10a–e and Additional File 13, Supplemental Table S7). The functional regulation networks of the *GhVQs* were constructed using the website of STRING11.0 with the module reference of *Arabidopsis* association, and the results revealed that the *GhVQs* participated in plant defense interaction networks, including WRKYs, MYB15, MPK4, AR781, CSN5B and SIGAs (Fig. 10f). Indeed, VQ proteins could interact with WRKYs and other TFs to defend against abiotic stresses in cotton.

## Discussion

In previous studies, the *VQ* family genes have been systematically analyzed in *Arabidopsis* [24], soybean [25], sunflower [31], rice [26], banana [43], maize [27], bamboo [44], *Cicer arietinum* [32], *Medicago truncatula* [32], and tobacco [19], and has shown to play significant roles in regulating growth, development processes, and responding to biotic and abiotic stresses [2]. Here, we complete a comprehensive analysis of the *VQ* family genes and explore their evolutional mechanism in *Gossypium* species.

### The expansion, duplication and structural characteristics of *VQs* in *Gossypium*

In this study, we analyzed the *VQs* of *G. hirsutum*, *G. barbadense.*, *G. raimondii*, *G. arboretum* and another 11 plant species, and found that the number of *VQs* in the genomes of 15 species was inconsistent with related to the size of their genomes. There are 89 *GhVQs*, 89 *GbVQs*, 45 *GaVQs*, and 45 *GrVQs*, respectively. The number of *Gossypium VQs* was higher than that in *cacao* (27 *TcVQs*) and in *Arabidopsis* (34 *AtVQs*), but the number of *GrVQs* and *GaVQs* was fewer than the *DzVQs* (Fig. 1 and Additional File 2, Supplemental Table S2). Previous studies have shown that diverse WGD events lead to the different sizes of plant genomes [45–47]. Our results indicated that *VQs* in these four *Gossypium* species were more likely to be proximal, tandem, and segmental genes, while the majority of *VQs* in rice [26] and *Arabidopsis* [24] are singleton genes. Through the analysis of the phylogenetic and structural features of the 15 plants VQ domains, the *VQs* could be divided into 10 clades. Group III could be expanded in the eudicots, particularly in the Mallow species (Fig. 2), while Groups III, IV, V, VI, VII, and X had no *VvVQs*, suggesting that these might have been lost in ancient genome duplication events.

Ten conserved motifs were also identified in the four *Gossypium VQs*. Motif 1 corresponded with the VQ-containing motif, which is widely found in angiosperms [3]. Previous studies have suggested that *VQs* have few introns in higher plants, being agreement with the results from *Gossypium VQs*. Only 3 *GhVQs*, 3 *GbVQs*, 14 *GrVQs*, and 3 *GaVQs* had multiple introns. Additionally, the motif compositions and intron contents of the *Gossypium* VQ proteins/genes in our study were consistent with the results of the phylogenetic analysis and the type of gene duplication. Collectively, our data speculated that *Gossypium VQs* might be affected by intronic evolution.

### *VQs* play important roles in abiotic stress signaling pathways

Previous reports have shown that *VQs* are involved in various endogenous and environmental signals, which consistent with their diverse roles in plant development and in the response to abiotic stresses [2, 5, 19, 24, 26, 32]. For example, *AtVQ08*, *AtVQ14*, *AtVQ17*, *AtVQ18* and *AtVQ22* are involved in modulation of seed development, chloroplast development, and plant growth. A proportion of the *GmVQs* [25], *PeVQs* [44], *VvVQs* [48], *CsVQs* [29], *HaVQs* [31], and *NtVQs* [19] also function in regulating the growth of different tissues at different developmental stages. In this study, most *GhVQs* and *GbVQs* were found to differentially expressed in the different tissues, including the ovule, fiber, anther, leaf, root, sepal and stem, suggesting that the *VQs* may play an important role in growth and development of *Gossypium* species (Fig. 7, Additional Files 6 and 7, Supplemental Fig. S6 and S7). Most *VQs* have been demonstrated to play important roles in responses to various abiotic stresses in plants [2, 3, 19, 27, 30, 32]. In our work, we assessed the expression levels of the *GhVQs* and *GbVQs* under salt, drought, cold, and heat stresses, and found that the majority of the *VQs* were up-regulated under drought, salt, and cold stress, or down-regulated under heat stress. These findings were similar to those of previous reports in *Arabidopsis* [5], rice [26], maize [27] and cabbage [28]. Also, the promoters of the *Gossypium VQs*, many cis-elements that were reported to exist in other abiotic stresses responsive genes were detected (Fig. 3), implying that the *VQs* in *Gossypium* are likely involved in response to various abiotic stresses, and that the response mechanisms maybe complex and diverse.

*VQs* has been reported to interact with WRKY TFs and to regulate a variety of physiological and biochemical processes and abiotic stress responses [6, 9, 11, 49, 50]. Here, by constructing co-expression and an *Arabidopsis* associated model, multiple *Gossypium VQs*, such as *GhVQ37*, *GhVQ59*, and *GhVQ83*, were predicted to interact with different WRKY TFs, implying that *VQs* act in stress tolerance through interacting with WRKYs. Moreover, we predicted some putative target sites of microRNAs in the *Gossypium VQs*. These microRNAs included miRNA156s and miR172, which serve important roles in various life processes of plants [40–42]. These results indicate that the *Gossypium VQs* are extensively involved in growth, development, and in response to stresses, and work together with WRKYs and microRNAs during these processes.

## Conclusions

In this study, using bioinformatics plus expression profiles, we identified and presented the structure, phylogenetic relationships, and tissue specificity of *VQ* family genes in four *Gossypium* species. Our data showed that the gene structure and motif coding regions were conserved across plants, and segmental, dispersed, and tandem duplications were the main reason for the expansion of the *VQs*. Cis-element and expression analyses indicated that the majority of *VQs* were activated in response to abiotic stress, and some of *VQs* were co-expressed with WRKYs and hybridized with the miRNAs involved in *Gossypium* growth, development, and abiotic stress. Our study could serve as a foundation for future exploration of the specific functions of *Gossypium VQs* in the abiotic stress responses and the interactions with WRKYs or microRNAs.

## Methods

### Identification and classification of *VQs* in plants

The latest versions of predicted proteomes of *G. raimondii* (https://phytozome.jgi.doe.gov/pz/portal.html#!info?alias=Org_Graimondii) [35], *G. arboreum* (ftp://bioinfo.ayit.edu.cn/downloads/) [34], *G. hirsutum*, and *G. barbadense* (https://ibi.zju.edu.cn/cotton/) [33] were used in this study. The genome data of other plants were obtained from the JGI database (https://www.phytozome.net) and National Center for Biotechnology Information (NCBI) database (https://www.ncbi.nlm.nih.gov/): *A. thanliana*, *V. vinifera*, *T. cacao*, *P. trichocarpa*, *P. dactylifera*, *O. sative*, *M. acuminate*, *D. zibethinus*, *C. papaya*, *B. rapacious*, and *A. trichopoda*. The pre-classified groups of these species were based on their phylogenetic relationships (https://www.timetree.org/) [51]. The VQ conserved domain (PF05678) was used as a query to scan the *Gossypium* species protein databases, and the *A. thaliana* VQ proteins were used as the queries to search against the above proteomes through the basic local alignment search tool (BLAST, v 2.10.0) (score value ≥0.0001 and E-value = 1 × 10^− 3^) [52] for each newly identified gene. The obtained putative VQ motif-containing sequences were confirmed in the NCBI Conserved Domain Database (https://www.ncbi.nlm.nih.gov/Structure/cdd/wrpsb.cgi) [53] and SMART database (https://smart.embl-heidelberg.de/) [54]. Then, the physical and chemical properties of the *VQ* family members, including amino acid length, mRNA length, MW, and pI, were analyzed using the online tools of the web site of softberry website (https://linux1.softberry.com/berry.phtml) and the ExPASy website (https://web.expasy.org/translate/) [55], and the relative results were plotted by the ggstatsplot (v 0.4.0) [56].

### Phylogenetic and synteny analysis of the *VQs* in plants

All the VQ motif-containing proteins from the four *Gossypium* and other 11 plant species were aligned using MAFFT (Multiple Alignment using Fast Fourier Transform, v 7.4.0.7) (L-INS-algorithms) [57] with default parameters, and conserved site sequences were selected by the Gblock (v 0.91b) software (https://molevol.cmima.csic.es/castresana/Gblocks_server.html) [58]. A phylogenetic tree was constructed using the IQ-TREE software (v 1.6.9) (https://www.iqtree.org/) [59] with the maximum likelihood method, and the substitution model was calculated with ModelFinder (intergraded in IQ-TREE; best-fit model: JTT + R5 chosen according to BIC). The obtained treefile was visualized using the ggtree (v 2.0.2) [60] and AI (Adobe Illustrator CS6).

The synteny and collinearity of duplication genes were analyzed in *Gossypium* species including *G. hirsutum* and *G. arboreum*, *G. hirsutum*, and *G. raimondii*; *G. barbadense* and *G. arboreum*; *G. barbadense* and *G. raimondii*; and *G. hirsutum* and *G. barbadense* using the modified MCScan algorithm of the MCScanX package (default parameters) (https://chibba.pgml.uga.edu/mcscan2/) [61]. All results were drawn using Circos (https://circos.ca/) [62].

### Analysis of chromosome location and gene regulatory elements

The chromosomal positions of all *VQ* members were determined using the gene transfer (gtf) format files of the reference genomes. The exon/intron structure of VQs were also extracted from the gtf files and displayed by the GSDS platform (http://gsds.cbi.pku.edu.cn/) [63]. Then, the MEME tool (http://meme-suite.org/) (the motif with 10 amino acids in length and E-value less than 1e^− 40^) [64] was used to detect the additional motifs of the proteins. With the combination of TBtools software (v 0.67361) [65], all domain motifs were compared among *VQ* genes to identify the group-specific signatures.

Approximately 2000 bp genomic sequences locating upstream (from − 2000 to − 1) of the *VQs* from start codons were extracted from the cotton genome, which was subsequently submitted to the online PlantCARE (http://bioinformatics.psb.ugent.be/webtools/plantcare/html/) [66] to determine the distribution of plant cis-acting regulatory elements. Moreover, the miRNA targets of the VQ members were predicted using the coding sequences (CDS) regions for complementary sequences by the psRNATarget server (http://plantgrn.noble.org/psRNATarget/analysis?function=2) [67] with default parameters, except maximum expectation (E) = 3.5. A total of 80 published miRNAs of *G. hirsutum* were selected.

### Expression profiles and co-expression networks

To determine the expression patterns of *VQs* in the two allotetraploid kinds of *Gossypium*, RNA-Seq data were obtained from the SRA database (PRJNA490626) [33], including those in 10 tissues (the petal, pistil, root, sepal, stem, torus, filament, leaf, anther, and bract), during ovule development (− 3, − 1, 0, 1, 3, 5, 10, 20, and 25 DPA ovule) and fiber development (10, 20, and 25 DPA), and under four different abiotic stresses (NaCl, PEG, cold, and hot), which were previously generated and analyzed by Hu et al. [33]. Raw RNA-seq reads were filtered using the SRAToolkit (v 2.9.2) / fastq-dump [68] and trimmed by Trimmomatic (v 0.3.9) [69] to generate clean reads. Then, the filtered clean RNA-seq reads were mapped to their respective reference genomes using the HISAT2 (v 2.1.0) [70], and the Sequence Alignment/Map format (sam) data was converted to Binary Alignment/Map (bam) data using the samtools (v 1.9) [71]. The transcript abundances of annotated genes were quantified using the stringtie (v 2.0) [72]. The gene expression data were obtained from the big databases by R base, and expression levels expressed as log2 (FPKM+ 1) and visualized using the pheatmap (v 1.0.12) [73]. The WGCNA (v 1.69) [74] and the STRING software (Searched Tool for the Retrieval of Interacting Genes/Proteins, http://string-db.org/) [75] were used for the analysis the co-expression networks, and the obtained results were displayed using the Cytoscape software (v 3.7.2) (http://www.cytoscape.org/) [76].

### Plant cultivation, RNA isolation, and RT-PCR analysis

The germinated TM-1 cotton seeds were grown in plastic pots filled with the mixture of soil vermiculite, and the artificial growth conditions were set at 28/22 °C, with a photoperiod of 16 h light/8 h darkness. Plants were separately subjected to 400 mM PEG or 400 mM NaCl. Three biological replicates were sampled at 0, 1, 3, 6, 12, 24, 36, or 72 h. All the samples were collected and frozen in liquid nitrogen, which was stored at − 80 °C until total RNA extraction.

Total RNAs of the above samples were isolated using the RNA prep Pure Plant Kit (Polysaccharides & Polyphenolics-rich, DP441) (TIANGEN, Beijing, China). The concentrations and integrities of the extracted RNA samples were measured and verified using a NanoDrop machine and 1% agarose gel electrophoresis, and the RNA samples were reversed transcribed into complementary DNA (cDNA) using the Mir-X TM MIRNA First-Strand Synthesis Kit (TaKaRa, Dalian, China). The qRT-PCR was performed using the Roche LightCycler 480 System (Roche, Germany). The qRT-PCR primers for the *GhVQs* and actin gene were listed in Supplemental Table S8. The reaction was set up in a total volume of 20 μL: 2 μL (200 ng) of cDNA, 0.4 μL of forwarding primer (10.0 μM), 0.4 μL of reverse primer (10.0 μM), 10 μL 2 × TransStart Top/Tip Green qPCR SuperMix, and 7.2 μL of nuclease-free water. The reaction procedure was completed under the following program: 94 °C for 30 s; 45 cycles of 94 °C for 5 s, 60 °C for 15 s, 72 °C for 10 s; and 4 °C to finish. The results were calculated using the 2^-ΔΔCt^ relative quantitative method.

## Supplementary Information

**Additional file 1: Table S1.** Summaries of *VQ* genes members in *Gossypium*.

**Additional file 2:** **Figure S1.** The physical and chemical properties of *VQs* in *Gossypium spp*. with violin illustration. **a, b, c, d, e, f** are the lengths of the transcripts, GC contents, exon numbers, protein lengths, PI values and MW values, respectively.

**Additional file 3: Table S2.** The number of *VQ* genes in the 15 plant genomes.

**Additional file 4: Figure S2.** The phylogenetic tree of *VQs* in *Gossypium spp.* compared with *A. thaliana*. **a** The phylogenetic tree of *VQ*s in *G. hirsutum* and *A. thaliana*. **b** The phylogenetic tree of *VQs* in *G. barbadense* and *A. thaliana*. **c** The phylogenetic tree of *VQs* in *G. raimondii* and *A. thaliana*. **d** The phylogenetic tree of *VQs* in *G. arboretum* and *A. thaliana*.

**Additional file 5: Figure S3.** The conserved motifs of *VQs*.

**Additional file 6: Figure S4.** The paralogs of *VQs* between *G. hirsutum* and another two diploid *Gossypium* species. The lines regarding orthologous gene pairs are colored by green and blue. The green lines are the pairs between *G. hirsutum* D-subgenome and *G. raimondii* genome, and the blue lines are the pairs between *G. hirsutum* A-subgenome and *G. arboretum* genome.

**Additional file 7: Table S3.** The list of paralogous *VQ* gene pairs in each of other examined species.

**Additional file 8: Figure S5.** The paralogs of *VQs* between *G. barbadense* and another two diploid *Gossypium* species. The lines regarding orthologous gene pairs are colored by green and blue. The green lines are the pairs between *G. barbadense* D-subgenome and *G. raimondii* genome; and the blue lines are the pairs between *G. barbadense* A-subgenome and *G. arboretum* genome.

**Additional file 9: Table S4.** The duplicated type of *VQs* in *Gossypium* spp. The 0 to 4 indicate the singleton, dispersed, proximal, tandem, WGD duplication types, respectively.

**Additional file 10: Table S5.** List of predicted known microRNA target sites of *VQ* transcripts in *Gossypium* species.

**Additional file 11: Figure S6.** Expression patterns of *GhVQs* in different tissues and under stresses in *G. hirsutum*.

**Additional file 12: Figure S7.** Expression patterns of *GbVQs* in different tissues and under stresses in *G. barbadense*.

**Additional file 13: Table S6.** Expression levels of *GhVQs* and *GbVQs*. The gene expression was determined by RNA-Seq data (FPKM).

**Additional file 14: Table S7.** Information regarding the Co-expression and STRING search results of the interaction networks of the *GhVQs*.

**Additional file 15: Table S8.** Primers used for qPCR analyses of *GhVQs*.

## Data Availability

All data supporting the conclusions of this article are included in the article and its additional files.
