# Supplementary figures and images for: A comprehensive analysis of cotton VQ gene superfamily reveals their potential and extensive roles in regulating cotton abiotic stress
Source: BMC Genomics. 2020 Nov 16;21:795. doi: 10.1186/s12864-020-07171-z (PMC7667805; doi:10.1186/s12864-020-07171-z)

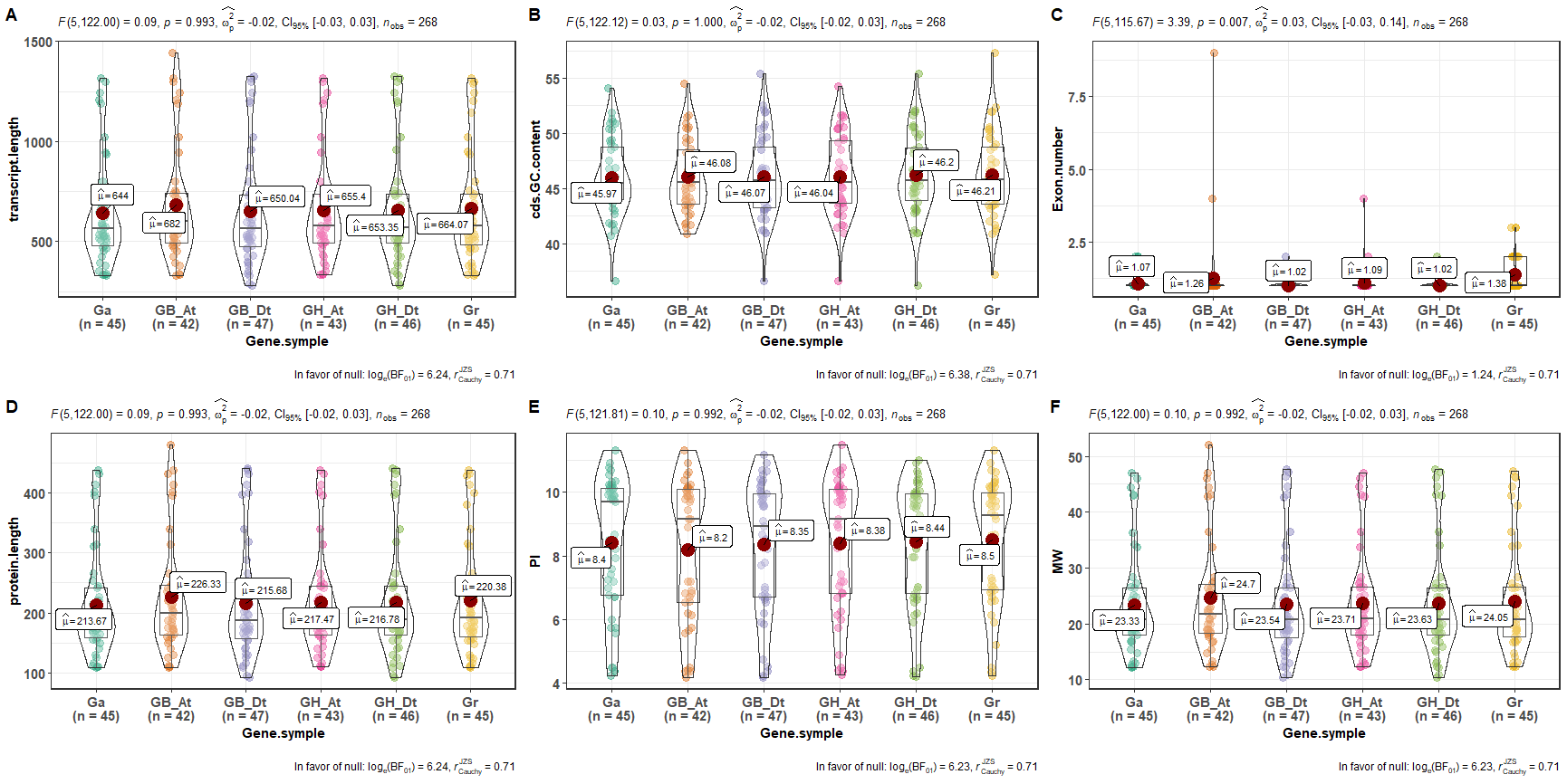

Supplement: Supplementary file 2 — Additional file 2: Figure S1. The physical and chemical properties of VQs in Gossypium spp. with violin illustration. a, b, c, d, e, f are the lengths of the transcripts, GC contents, exon numbers, protein lengths, PI values and MW values, respectively. [file 12864_2020_7171_MOESM2_ESM.tif]

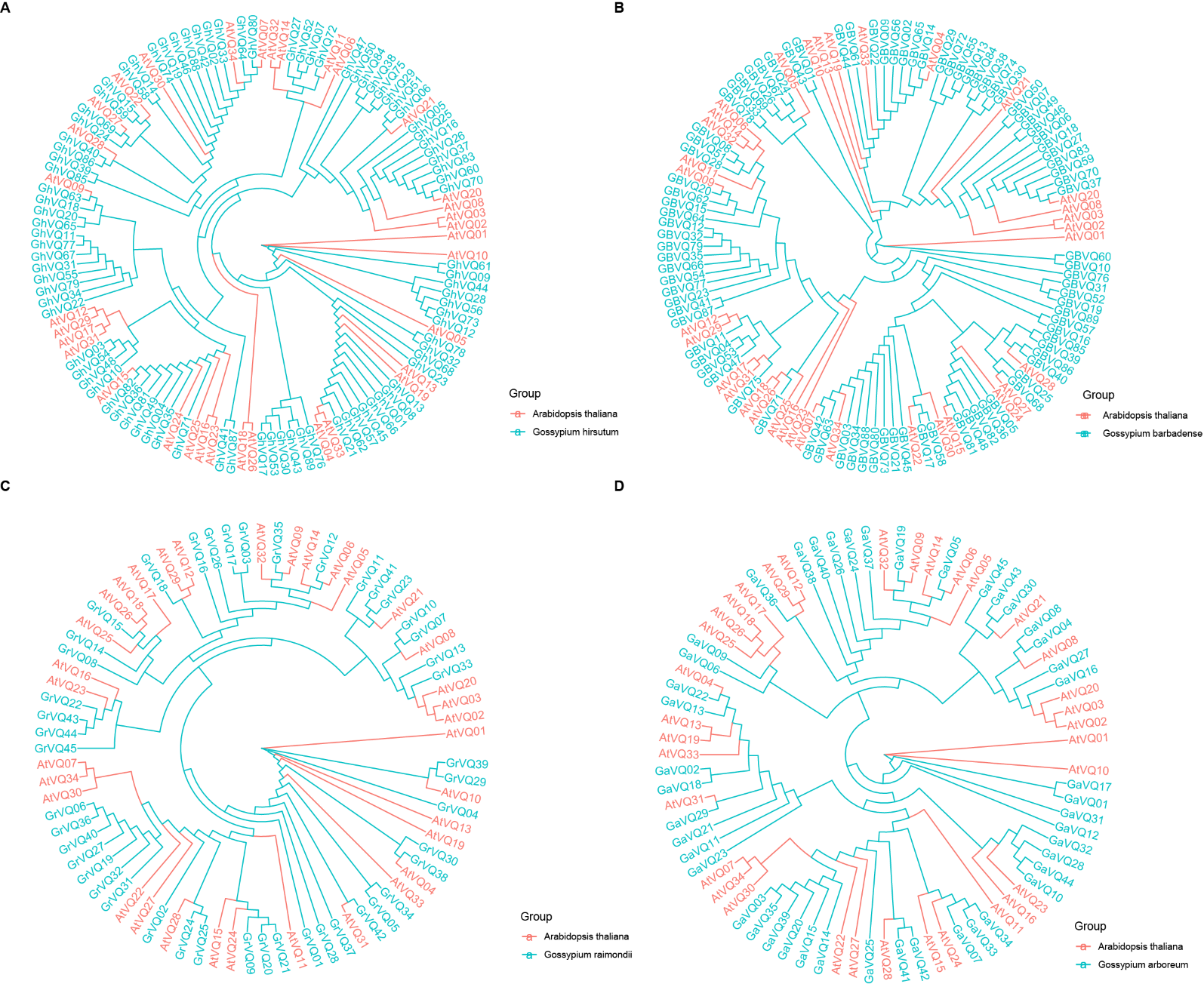

Supplement: Supplementary file 4 — Additional file 4: Figure S2. The phylogenetic tree of VQs in Gossypium spp. compared with A. thaliana. a The phylogenetic tree of VQs in G. hirsutum and A. thaliana. b The phylogenetic tree of VQs in G. barbadense and A. thaliana. c The phylogenetic tree of VQs in G. raimondii and A. thaliana. d The phylogenetic tree of VQs in G. arboretum and A. thaliana. [file 12864_2020_7171_MOESM4_ESM.tif]

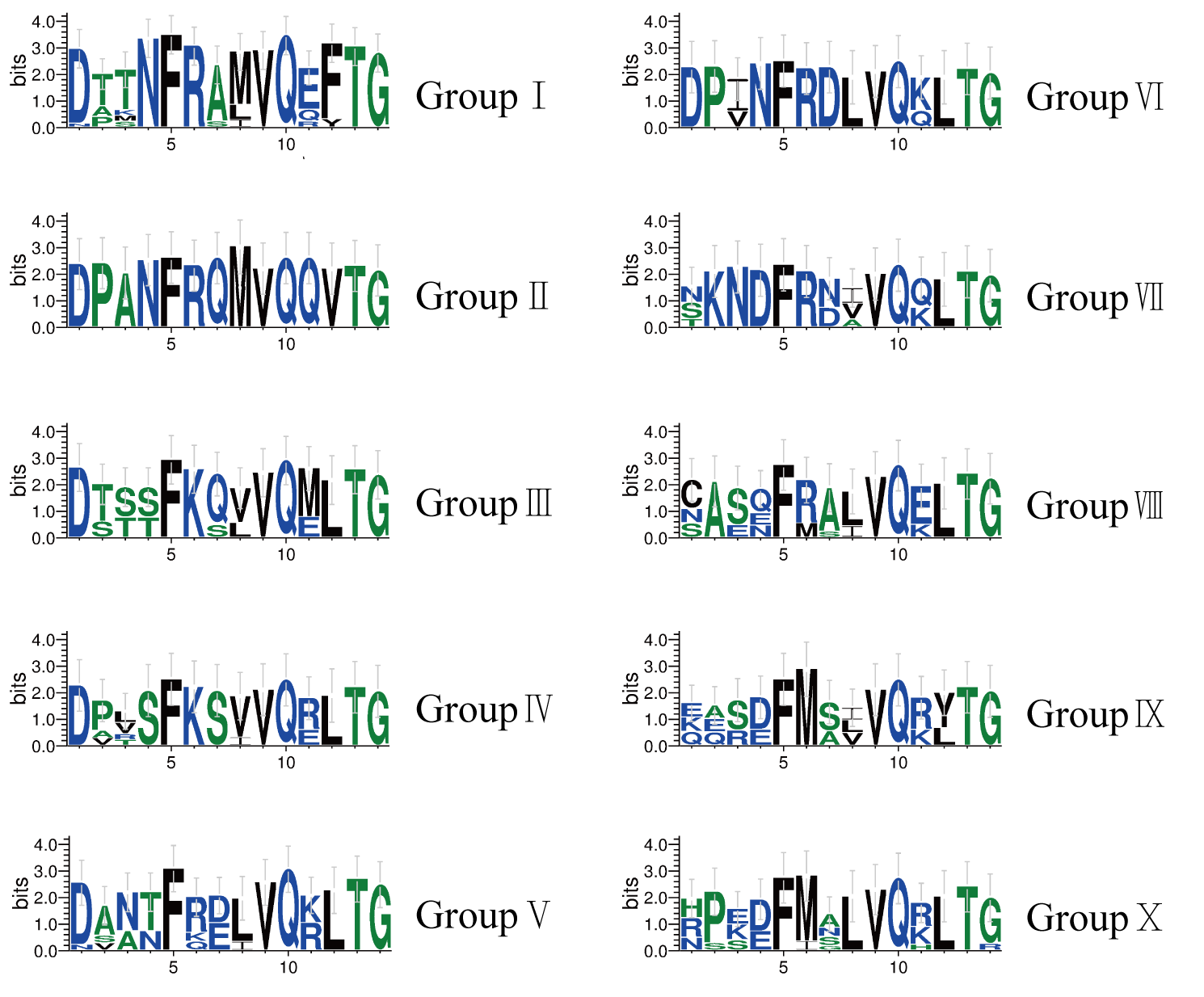

Supplement: Supplementary file 5 — Additional file 5: Figure S3. The conserved motifs of VQs. [file 12864_2020_7171_MOESM5_ESM.tif]

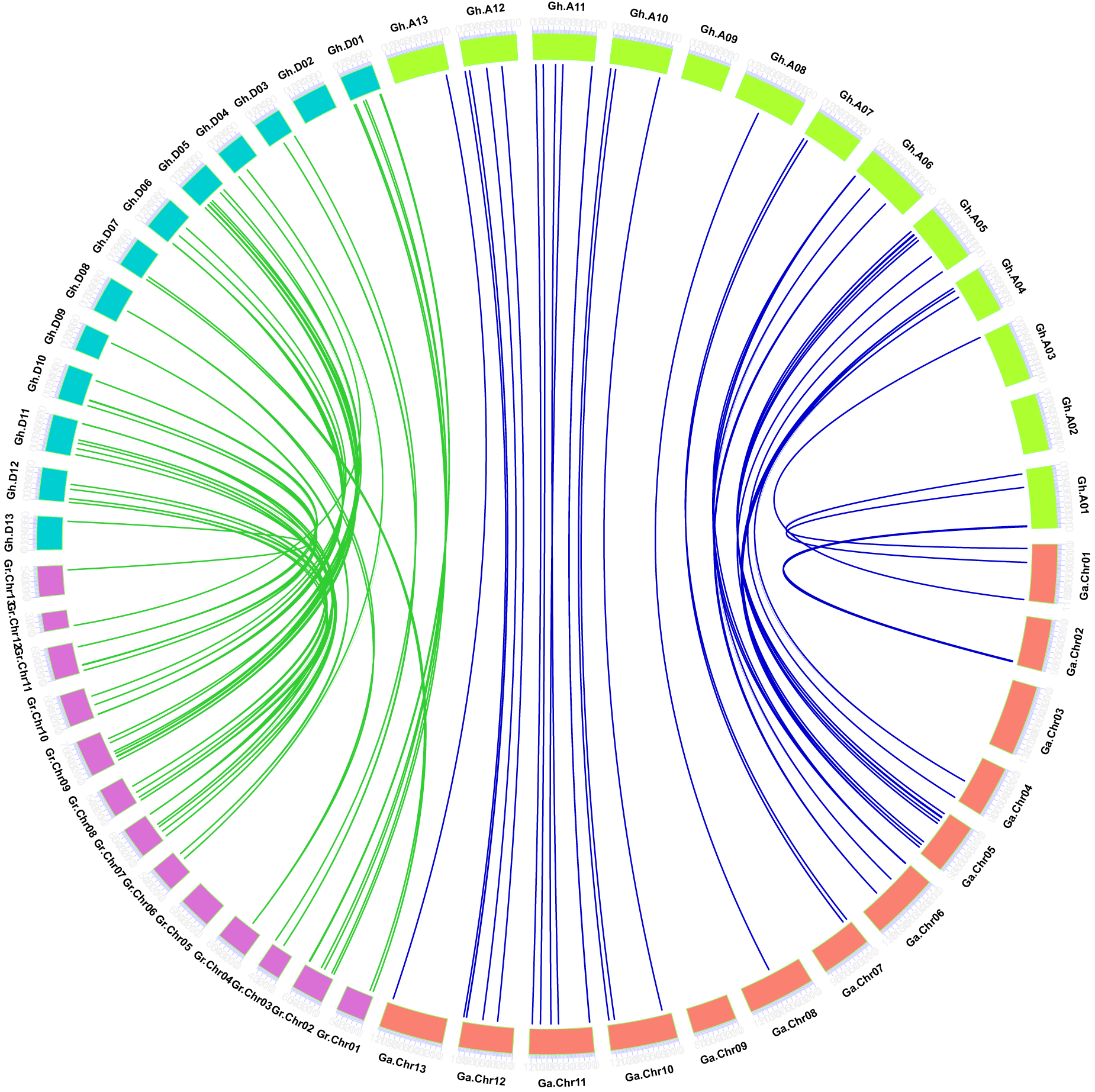

Supplement: Supplementary file 6 — Additional file 6: Figure S4. The paralogs of VQs between G. hirsutum and another two diploid Gossypium species. The lines regarding orthologous gene pairs are colored by green and blue. The green lines are the pairs between G. hirsutum D-subgenome and G. raimondii genome, and the blue lines are the pairs between G. hirsutum A-subgenome and G. arboretum genome. [file 12864_2020_7171_MOESM6_ESM.tif]

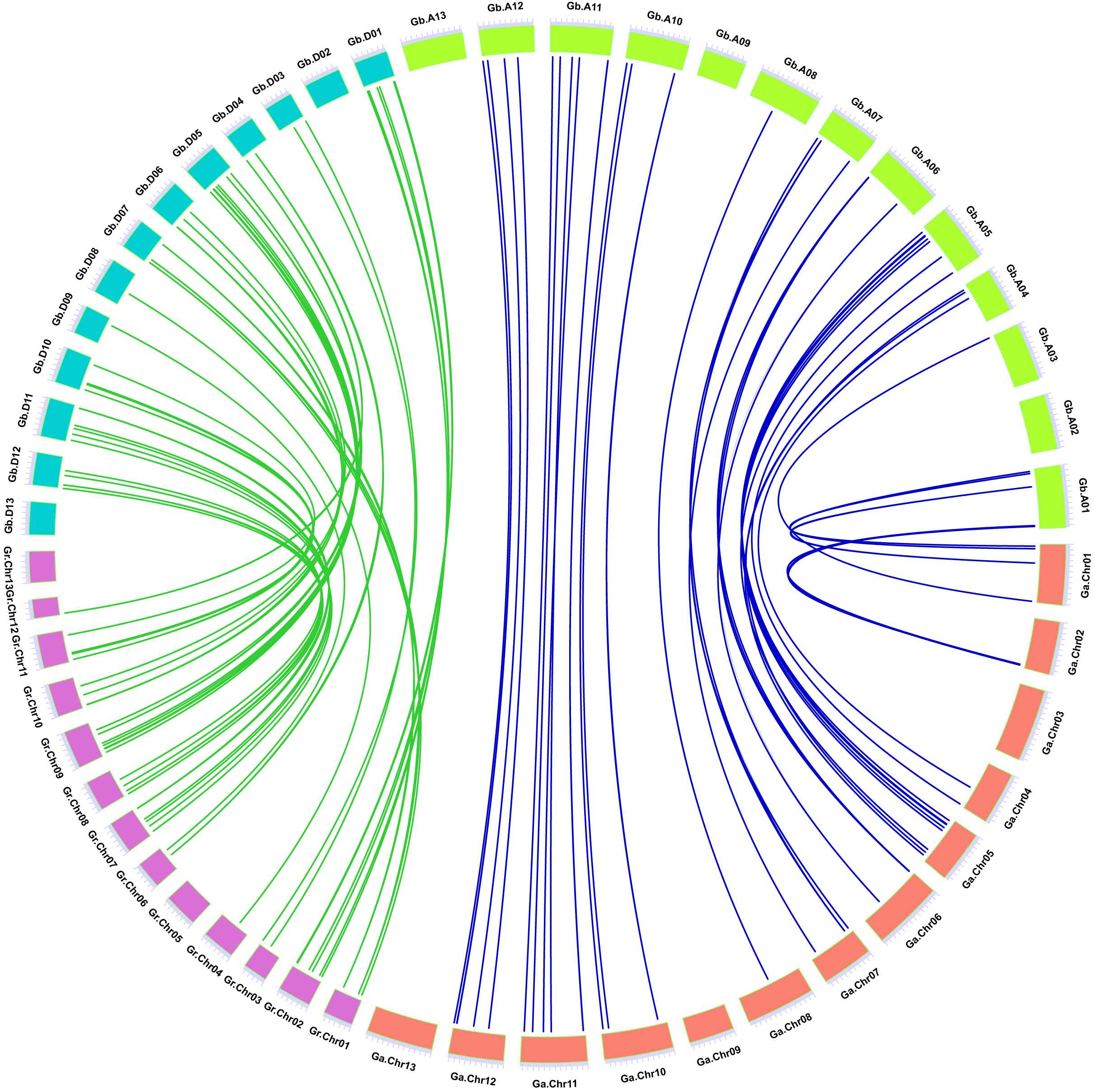

Supplement: Supplementary file 8 — Additional file 8: Figure S5. The paralogs of VQs between G. barbadense and another two diploid Gossypium species. The lines regarding orthologous gene pairs are colored by green and blue. The green lines are the pairs between G. barbadense D-subgenome and G. raimondii genome; and the blue lines are the pairs between G. barbadense A-subgenome and G. arboretum genome. [file 12864_2020_7171_MOESM8_ESM.tif]

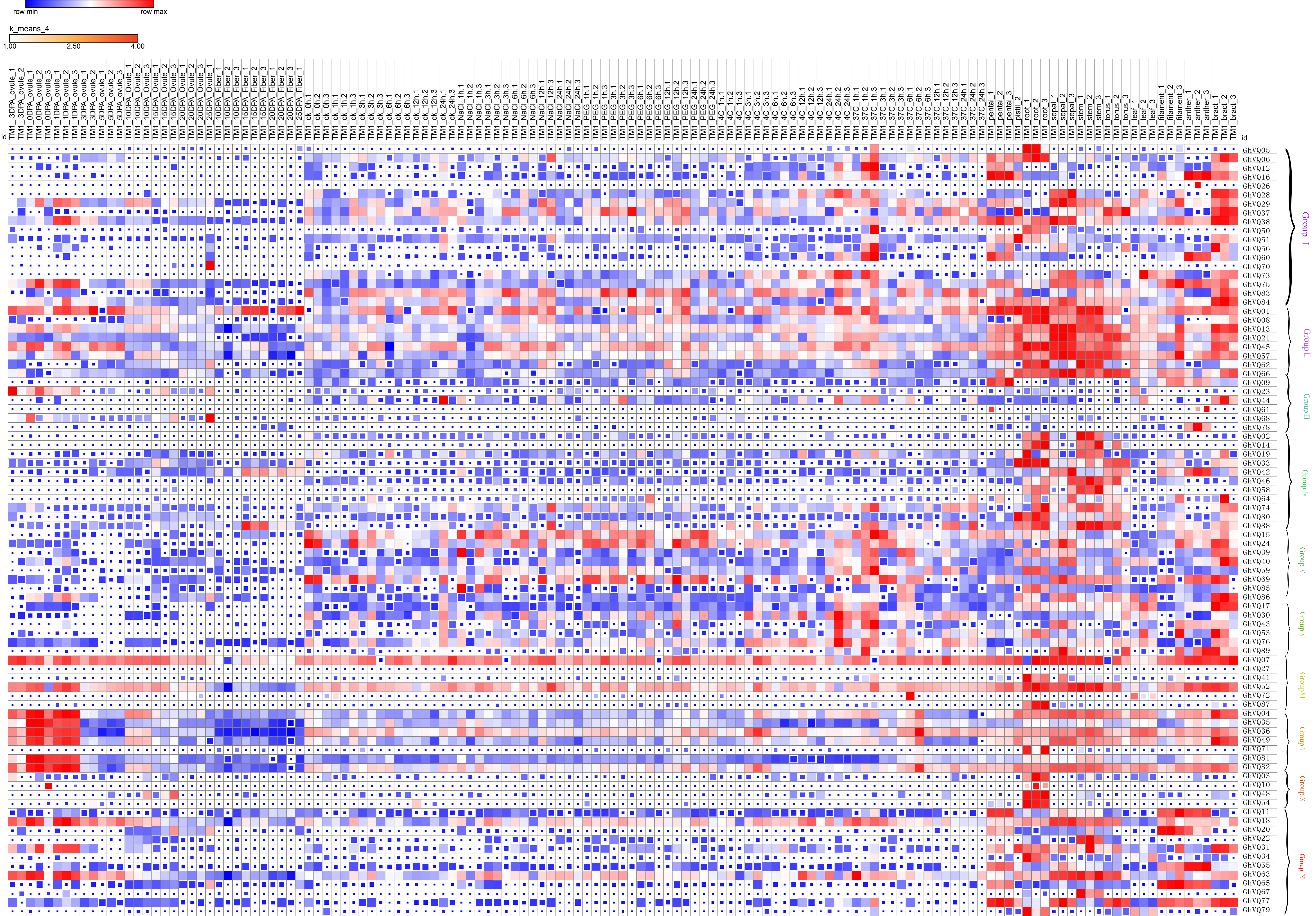

Supplement: Supplementary file 11 — Additional file 11: Figure S6. Expression patterns of GhVQs in different tissues and under stresses in G. hirsutum. [file 12864_2020_7171_MOESM11_ESM.pdf]

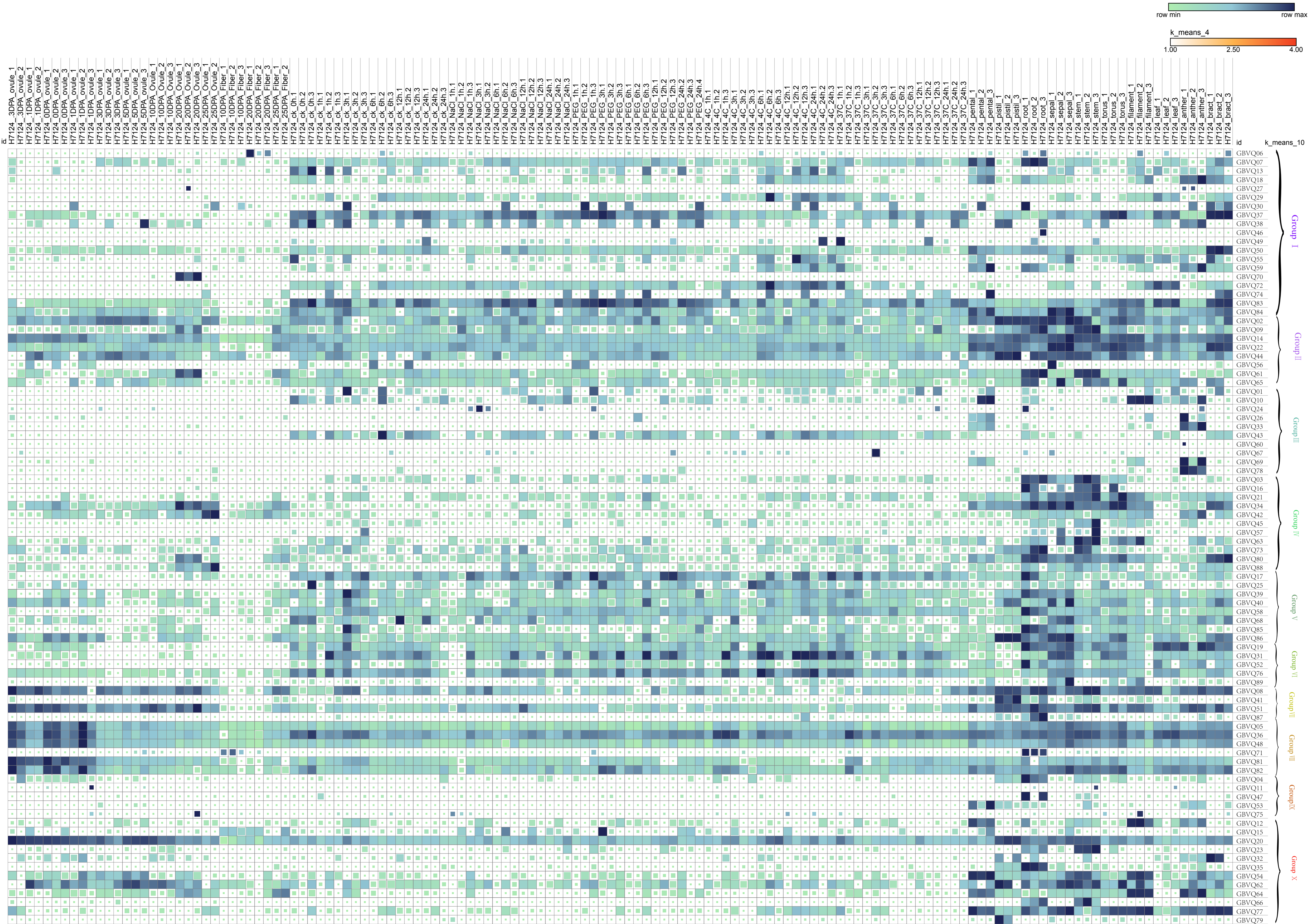

Supplement: Supplementary file 12 — Additional file 12: Figure S7. Expression patterns of GbVQs in different tissues and under stresses in G. barbadense. [file 12864_2020_7171_MOESM12_ESM.pdf]
